# A Simple Microwave-Assisted Method for the Determination of Boron, Sulfur, Bromine, Chlorine, and Iodine in Whole Blood by ICP-QQQ for Biomonitoring Applications

**DOI:** 10.3390/toxics14100859

**Published:** 2026-09-28

**Authors:** Lucas Schmidt, Alma Malibekova, Thieli Schaefer Nunes, Kayla Peterson, Rosalind Wright, Julio Alberto Landero-Figueroa

**Affiliations:** 1 Department of Environmental Medicine, Icahn School of Medicine at Mount Sinai, New York, NY 10029, USA; lucas.schmidt@mssm.edu (L.S.); thieli.nunes@mssm.edu (T.S.N.); kayla.peterson@mssm.edu (K.P.); rosalind.wright@mssm.edu (R.W.); 2 Department of Chemical and Environmental Engineering, University of Cincinnati, Cincinnati, OH 45219, USA; malibeaa@mail.uc.edu

**Keywords:** halogens, non-metals, boron, bromine, chlorine, iodine, sulfur, ICP-QQQ, biofluid, blood

## Abstract

The simultaneous determination of non-metal elements in whole blood remains analytically challenging because of the complex biological matrix and the wide concentration range. This study describes a simple microwave-assisted alkaline extraction method for the determination of boron, sulfur, bromine, chlorine and iodine in human whole blood by triple quadrupole inductively coupled plasma mass spectrometry. The proposed procedure requires only 100 µL of whole blood, which is processed directly in a disposable 5 mL polypropylene vial that is subsequently introduced into the autosampler, eliminating sample transfer steps and reducing handling and contamination risks. A single instrumental configuration was applied for all analytes using oxygen as the reaction/collision gas, while the formation of ^35^Cl^18^O^+^ was deliberately employed to attenuate the chlorine signal, allowing the simultaneous determination of major- and trace-level elements without detector saturation. The proposed method showed satisfactory precision (RSD < 15%), agreement with reference and assigned values, spike recoveries, limited effects of matrix load, and analytical solution stability for at least 24 h at room temperature. Limit of detection of whole-blood were 6.03 µg L^−1^ for boron, 0.134 mg L^−1^ for sulfur, 24.4 mg L^−1^ for chlorine, 6.26 µg L^−1^ for bromine, and 0.944 µg L^−1^ for iodine, providing suitable sensitivity for concentrations typically encountered in human whole blood. Overall, the method provides a simple, robust, and high-throughput approach for routine biomonitoring of non-metals in whole blood.

## 1. Introduction

Human biomonitoring has become an important tool for assessing nutritional status, environmental and occupational exposures, and certain disease-related metabolic alterations. Elemental analysis of whole blood can provide valuable information on the physiological status and exposure of individuals, as blood serves as a major transport medium for minerals, electrolytes, and trace elements [1]. The application of elemental blood analysis in large-scale epidemiological and clinical studies requires reliable, high-throughput, and multi-element analytical methods. In this context, the determination of B, S, Cl, Br, and I can provide complementary information related to nutrition, essential physiological processes, and environmental or toxicological exposures [2].

Cl is the major extracellular anion, representing 70% of total anions within the body, chloride is a vital electrolyte responsible for regulating acid–base homeostasis, osmotic pressure, fluid distribution, and muscle activity [3,4]. Additionally, chloride is essential for gastrointestinal health, directly facilitating protein digestion, adsorption of micronutrients, and the maintenance of gastric acidity [4]. Alterations in chloride levels are associated with metabolic disorders such as hypochloremic metabolic alkalosis and hyperchloremic metabolic acidosis [3,5]. Although historically overshadowed by sodium in clinical guidelines, chloride has recently emerged as a critical and independent prognostic marker in cardiovascular disease, particularly in heart failure [6,7,8]. Hypochloremia has been identified as a strong predictor of adverse outcomes, diuretic resistance, and increased mortality in heart failure patients [6,7,8].

Iodine is essential for the biosynthesis of thyroid hormones, which regulate cellular metabolism, growth, and fetal neurodevelopment [9,10]. Both chronic iodine deficiency and excessive intake can result in adverse health effects, including goiter, impaired neurodevelopment, and thyroid dysfunction [9,11,12]. Subclinical iodine deficiency has become the focus of several studies in pregnancy cohorts, particularly as traditional dietary sources of iodine, such as milk and iodized salt, are increasingly reduced or replaced [13,14,15]. Br, in contrast, was historically considered a non-essential element of primarily toxicological relevance, with excessive exposure associated with neurological, gastrointestinal, and dermatological effects [16]. However, recent research uncovered that bromine plays an essential function in the formation of sulfilimine crosslinks in collagen IV scaffolds, a process critical for tissue development and basement membrane architecture [17]. Because of its chemical similarity to I, excessive Br exposure may also interfere with I metabolism and thyroid homeostasis [18].

Although the essentiality of B in humans remains under study, increasing evidence indicates that B is a bioactive trace element involved in several physiological processes. B is a metalloid; for simplicity, it is included within the term “non-metals” throughout this study. B has been shown to influence the severity of arthritis, osteoporosis or bone fractures, facilitate hormone action, and support central nervous system function [19]. A clinical study evaluating a B-rich diet reported significant reductions in serum total, LDL, and VLDL cholesterol and triglycerides, as well as body weight, body fat, and body mass index, suggesting a potential role of B in lipid and energy metabolism [20]. On the other hand, sulfur is a major essential element present in some amino acids and proteins, including abundant blood proteins such as albumin. It plays important roles in cellular signaling, redox homeostasis, metabolism, and detoxification processes [21,22].

Despite their biological relevance, the determination of B, S, Cl, Br, and I within a single whole blood sample presents substantial analytical challenges. Conventional approaches for these elements frequently rely on separate or analyte-specific techniques, including ion-selective electrodes [23], coulometry [24], ion chromatography [25], inductively coupled plasma optical emission spectroscopy (ICP-OES) [26]. Moreover, these elements occur across several orders of magnitude in whole blood, ranging from trace concentrations of B and I to sub-percentage content of S and Cl. Their determination in the same analytical solution is further complicated by the protein and lipid rich blood matrix, spectral interferences, potential analyte losses, memory effects, and the limited compatibility of some sample preparation procedures with plasma-based techniques [2,27].

Inductively coupled plasma mass spectrometry (ICP-MS) is widely employed for elemental biomonitoring because of its high sensitivity, multi-element capability, and analytical throughput [1,28,29,30,31,32]. Nevertheless, reliable determination of non-metals in complex biological matrices depends strongly on the sample preparation procedure [2]. Conventional acidic digestion using concentrated oxidizing reagents can effectively decompose biological matrices but may be unsuitable for halogens because volatile species can be lost during sample preparation [2,33]. Alkaline extraction provides an attractive alternative because halogens generally exhibit improved stability under alkaline conditions, reducing the potential for volatilization losses [2,33,34]. Furthermore, many established approaches are optimized for individual elements or limited groups of analytes rather than for the determination of multiple non-metals within a single analytical workflow. For large-scale biomonitoring studies, where sample volume, throughput, contamination control, reagent consumption, and waste generation are important considerations, a simplified procedure capable of accommodating both trace- and major-level non-metals would therefore be advantageous. Microwave-assisted alkaline extraction in disposable vessels offers the possibility of combining the favorable stability of non-metals, particularly halogens, under alkaline conditions with controlled heating, low sample and reagent requirements, and reduced sample manipulation.

Therefore, the aim of this study was to develop and establish a simple, robust, microwave-assisted alkaline extraction method coupled with ICP-MS/MS for the simultaneous determination of B, S, Cl, Br, and I in whole blood samples. The proposed method requires only 100 µL of sample, enables direct analysis after extraction, combines trace and major elements in a single analytical run, employs oxygen reaction chemistry for S and Cl, and uses the low-abundance ^18^O isotope to control chlorine sensitivity while maintaining pulse-mode detection for all analytes. The analytical performance of the method was thoroughly evaluated, demonstrating its suitability for routine determination of B, S, Cl, Br, and I in whole blood for clinical and biomonitoring applications.

## 2. Materials and Methods

### 2.1. Instrumentation

A microwave-assisted extraction (MAE) system (Multiwave 5000, Anton Paar, Graz, Austria) equipped with an infrared temperature monitoring system and a 64MG5 rotor for micro-vessels was used for sample preparation. Disposable 5 mL polypropylene (PP) vials (4150-870-001-9, Labcon, Petaluma, CA, USA) were employed as the extraction vessels in the 64MG5 rotor throughout this study.

Elemental determinations were performed using an Agilent 8900 triple quadrupole inductively coupled plasma mass spectrometer (ICP-MS/MS) (Agilent Technologies, Santa Clara, CA, USA) equipped with a MicroMist 400 concentric nebulizer, a Peltier-cooled double-pass spray chamber, a quartz torch fitted with a 2.5 mm i.d. quartz injector, Pt sampler and skimmer cones, an X-lens ion optics system, and an SPS 4 autosampler. Moreover, O_2_ was used as a reaction/collision gas in the ICP-MS to remove and minimize any possible interferences such as ^16^O^16^O on ^32^S or ^16^O^17^O, on ^33^S, ^16^O^18^O^1^H on ^35^Cl, ^40^Ar^39^K and ^40^Ar^38^Ar^1^H on ^79^Br or ^113^In^14^N, ^115^In^12^C, ^126^Te^1^H and ^87^Sr^40^Ar and ^111^Cd^16^O on ^127^I. The latter interferences are unlikely to be encountered at significant levels in biological matrices but may become relevant in matrices containing high concentrations of their precursor elements, such as certain inorganic matrices [33,35,36,37].

Instrument operating conditions are summarized in Table 1.

### 2.2. Chemicals and Materials

Ultrapure water (18.2 MΩ cm, Milli-Q, Millipore, Bedford, MA, USA), 21% (*w*/*w*) ammonia solution (TraceMetal™ grade, Fisher Chemical, Fairlawn, NJ, USA), disodium ethylenediaminetetraacetate dihydrate (EDTA-2Na, Sigma-Aldrich, Burlington, MA, USA), and 25% (*w*/*w*) tetramethylammonium hydroxide (TMAH) solution (TCI America, Portland, OR, USA) were used to prepare all analytical solutions.

External calibration standards were prepared from commercially available single-element reference standards of boron (1000 mg L^−1^, Inorganic Ventures, Christiansburg, VA, USA), sulfur (10,000 mg L^−1^, Ricca Chemical, Arlington, TX, USA), chlorine (70,000 mg L^−1^, Sigma-Aldrich, St. Louis, MO, USA), bromine (1000 mg L^−1^, HPS, North Charleston, SC, USA), and iodine (1000 mg L^−1^, TraceCERT^®^, Merck, Darmstadt, Germany).

Internal standard (ISTD) solutions were prepared from commercially available stock solutions of iridium (1000 mg L^−1^) and antimony (100 mg L^−1^) (Inorganic Ventures, Christiansburg, VA, USA). These elements were added to all calibration standards, quality control solutions, blanks, and samples at final concentrations of 40 µg L^−1^ Ir and 200 µg L^−1^ Sb. Ultrapure water and a 240 mmol L^−1^ ammonia solution were used as rinse solutions between samples to minimize carryover. The selection of the ISTD and the rinse program was based on our recently published method for the determination of Cl and Br in urine and serum, where their suitability was previously demonstrated [33].

Calibration standards were prepared at seven concentration levels within the following ranges: 0.5–500 µg L^−1^ for B and Br, 0.05–50 µg L^−1^ for I, 0.05–50 mg L^−1^ for S, and 0.5–500 mg L^−1^ for Cl. For each analyte, at least five calibration levels were selected for regression based on the concentration range observed in the samples, thereby providing a calibration range more representative of the concentrations encountered during analysis. Detailed calibration parameters are provided in the Supplementary Materials (Table S1). All calibration standards were prepared in a solution containing 0.5% (*w*/*v*) TMAH, 0.1% (*w*/*v*) EDTA-2Na, and the corresponding internal standards. Based on preliminary method development, Sb and Ir were selected as the internal standards because they provided the most consistent correction of instrumental fluctuations and matrix-related effects throughout the analytical procedure. Sb was selected as the primary internal standard, and all results presented in this study were obtained using Sb for signal normalization. Ir was included as an alternative internal standard to provide additional flexibility in case the Sb is unsuitable for a particular sample due to unexpected elevated endogenous concentrations of Sb or unexpected analytical interferences. A comparison of Sb and Ir as internal standards is provided in the Supplementary Materials (Section S2).

Whole blood proficiency testing (PT) materials obtained from the Quebec Multielement External Quality Assessment Scheme (QMEQAS) (*n* = 6) were used as whole blood samples to evaluate the analytical performance of the proposed method.

Method accuracy was further assessed using NIST SRM 1577c (Bovine Liver), a certified reference milk powder material (CRM-MP, High-Purity Standards, Charleston, SC, USA), in-house quality control samples (QC-1 and QC-2), and spike recovery experiments. Samples were fortified with B, Br, Cl, I, and S at concentrations of 1.75 mg L^−1^, 1 mg L^−1^, 1000 mg L^−1^, 0.1 mg L^−1^, and 750 mg L^−1^, respectively. Also, assigned values for boron and bromine in NIST SRM 1577c were obtained from the 2025 Wadsworth Center Interlaboratory Study of Trace Element Analysis in Soft Tissues, with permission from the Wadsworth Center.

The stability of prepared analytical solutions was evaluated after storage for more than 24 h at room temperature (approximately 20 °C) and compared with freshly prepared extracts analyzed within 8 h after preparation.

### 2.3. Microwave-Assisted Alkaline Extraction

An aliquot of 100 µL of whole blood was transferred directly into a disposable 5 mL polypropylene (PP) vial. Subsequently, 700 µL of ultrapure water, 100 µL of 25% (*w*/*w*) TMAH solution, and 100 µL of a diluent containing 5% (*w*/*w*) EDTA-2Na, 50 mmol L^−1^ ammonia, 2 mg L^−1^ Ir, and 10 mg L^−1^ Sb were added to the same vial. The vial was immediately capped and vortex-mixed for approximately 10 s prior to microwave-assisted extraction (MAE).

The MAE was carried out using a maximum temperature of 90 °C with a heating ramp of 10 min, followed by a holding time of 15 min. After completion of the extraction cycle, the samples were allowed to cool inside the microwave system (until reaching below 50 °C). During the heating program, the microwave power was adjusted according to the number of vials in the rotor to ensure an appropriate heating rate, with the maximum temperature reached between 75% and 100% of the total ramp time, while maintaining uniform heating distribution across all samples.

Following extraction, each sample was quantitatively diluted to a final volume of 5 mL with ultrapure water and homogenized prior to ICP-MS analysis. Method blanks were prepared and processed using the same protocol, replacing the blood sample with ultrapure water.

The MAE conditions were selected based on preliminary experiments and previous TMAH-based procedures reported for biological matrices. The maximum temperature was set at 90 °C, consistent with previously reported alkaline extraction procedures using TMAH for biological fluids and tissues prior to ICP-MS analysis [38,39,40,41,42,43].

For each microwave extraction batch, NIST SRM 1577c (Bovine Liver) was processed as a quality control material using 25 mg of sample instead of 100 µL of whole blood.

### 2.4. ICP-MS Analysis

The prepared extracts were analyzed directly by ICP-MS/MS without any additional sample preparation. B, S, Cl, Br, and I were determined in a single analytical run using oxygen as the reaction gas. Quantification was performed by external calibration and internal standard correction. Calibration curves were fitted using weighted (1/y) linear regression without forcing the regression through the origin. Moreover, B was quantified using ^11^B because of its higher natural abundance and sensitivity. The ^10^B isotope was retained as an optional monitored isotope to verify the absence of significant matrix-dependent spectral interferences and isotopic enrichment. The results obtained for ^10^B are provided in the Supplementary Materials (Tables S2–S4). The monitored isotopes, reaction product ions, internal standards, and instrumental operating conditions are summarized in Table 1.

Method detection (LOD) and quantification limits (LOQ) were calculated as three and ten times, respectively, the standard deviation obtained from 10 independently prepared method blanks.

Method precision was assessed by repeated analysis of proficiency testing materials, certified reference materials, and in-house quality control analytical solutions. Accuracy was evaluated by comparison with the reference values and by recovery experiments performed using spiked samples. Finally, the stability of prepared extracts was assessed by comparing analytical results obtained immediately after preparation and after 24 h of storage at room temperature.

Furthermore, one of the main advantages of the proposed procedure is that the same disposable polypropylene vial used for microwave-assisted extraction is directly compatible with the ICP-MS autosampler. Consequently, no post-extraction transfer step is required prior to analysis, minimizing sample handling, reducing the risks of contamination and analyte loss, and improving the overall robustness and throughput of the analytical workflow.

## 3. Results and Discussion

### 3.1. Analytical Strategy for the Determination of Non-Metals in Whole Blood by ICP-MS/MS

The main analytical challenge addressed in this study was to establish a single ICP-MS/MS method capable of determining B, S, Cl, Br, and I under the same instrumental conditions (single tune mode). These analytes differ in their physiological concentrations, ionization characteristics, and susceptibility to spectral interferences. Nevertheless, all 5 elements were successfully determined using a single instrumental tune, O_2_ as the collision/reaction gas, simplifying the analytical workflow while improving robustness, analytical frequency and sample throughput. Although only S and Cl were monitored as oxide product ions, O_2_ was used as the common cell gas for the determination of all analytes. Under these conditions, B, Br, and I were successfully determined as atomic ions without compromising analytical performance.

Sulfur was quantified using ^33^S in O_2_ reaction mode instead of the more abundant ^32^S isotope because the resulting ^33^S^16^O^+^ product ion provided sufficient sensitivity while reducing signal intensity, thereby improving measurement robustness and minimizing the risk of detector saturation. A similar strategy was adopted for Cl. Unlike Br and I, Cl is present in whole blood at concentrations several orders of magnitude higher than the other halogens. Consequently, monitoring ^35^Cl^16^O^+^ would restrict the concentration range that can be measured in pulse mode, whereas monitoring ^35^Cl^18^O^+^ extends the measurable Cl range while maintaining pulse-mode detection. To overcome this limitation, Cl was monitored as ^35^Cl^18^O^+^, taking advantage of the low natural abundance of ^18^O (approximately 0.2%) in the oxygen gas. Rather than maximizing sensitivity, this approach intentionally attenuated the product-ion signal to a level suitable for routine ICP-MS/MS analysis while maintaining adequate sensitivity for Cl determination in whole blood. This strategy enabled the determination of Cl together with B, S, Br, and I under identical instrumental conditions without detector saturation.

The deliberate use of ^18^O isotope to regulate detector response has received little attention in ICP-MS/MS applications. Rather than maximizing signal intensity, the formation of ^35^Cl^18^O^+^ provides a controlled attenuation of the analytical signal (approximately 300-fold lower sensitivity than ^35^Cl^16^O^+^) while maintaining sufficient sensitivity for the determination of Cl in whole blood (see Supplementary Materials, Table S7). This simple approach effectively extends the dynamic range of the method, enabling Cl to be determined together with B, S, Br, and I under identical instrumental conditions while maintaining pulse-counting detection for all analytes and limiting detector exposure to excessively high-count rates. To the best of our knowledge, this is the first report describing the deliberate exploitation of the natural abundance of ^18^O in O_2_ reaction gas for analytical signal attenuation in ICP-MS/MS.

An additional factor contributing to the robustness of the proposed method was the use of plasma option gas (5% of 20% O_2_ in Ar). In addition to the organic content of whole blood, the analytical solutions contained approximately 2.96 g L^−1^ of carbon originating from the analytical reagents, primarily 0.5% TMAH and 0.1% EDTA-2Na, resulting in a substantial carbon load even during the analysis of calibration standards. Based on observations during preliminary method development, the plasma option gas was introduced to facilitate oxidation of the residual organic matrix. Operation without the option gas resulted in greater signal variability and visibly greater carbon deposition at the interface, whereas its introduction reduced carbon deposition on the sampler and skimmer cones and improved signal stability. Therefore, the plasma option gas was maintained throughout the analytical sequence to improve instrumental stability and minimize interface contamination, particularly during the analysis of large sample batches.

The alkaline extraction solution containing TMAH and EDTA also contributed to the overall analytical performance. Alkaline media have been widely employed for the determination of halogens because they improve analyte stability while minimizing volatilization losses [2,33]. In addition, EDTA complexes multivalent cations naturally present in whole blood, preventing precipitation and reducing the possibility of analyte co-precipitation or salt deposition within the sample introduction system [33]. Similar alkaline reagent compositions have previously demonstrated excellent performance for the determination of halogens in biological fluids, including urine and serum, particularly with respect to carryover reduction and long-term instrumental stability [33]. Based on this previous evaluation, the same rinse strategy was adopted in the present method, and no deposition-related problems were observed throughout the analytical sequences.

Among the analytes investigated in this method, Br warranted additional experimental evaluation of potential spectral interference because ^40^Ar^39^K^+^ may contribute to the signal at m/z 79 and K is present at relatively high concentrations in whole blood. In contrast, the potential interferences listed for ^127^I^+^ involve elements that are not expected to occur at sufficiently high concentrations in whole blood to produce substantial contributions from the corresponding polyatomic species under the conditions employed in this study. This interpretation is also supported by the satisfactory agreement obtained for I in the whole-blood proficiency testing materials. Therefore, the potential contribution of this K-derived polyatomic interference to ^79^Br^+^ determination was experimentally evaluated. K may also be further introduced through the use of K_2_EDTA-containing blood collection tubes. Whole-blood samples were therefore fortified with increasing concentrations of K to evaluate the selectivity of ^79^Br^+^ determination under the O_2_ cell conditions employed in this method. No measurable K-related contribution to the ^79^Br^+^ signal was observed, even after the addition of 3000 mg L^−1^ K to the whole-blood sample. This additional K load resulted in a total K concentration substantially higher than would be expected from endogenous blood K and routine K_2_EDTA anticoagulation. Detailed experimental conditions and results are provided in the Supplementary Materials (Table S8).

### 3.2. Performance of Analytical Method

The analytical solution (A-) and sample (S-) based LODs and LOQs obtained for B, S, Cl, Br, and I are summarized in Table 2. Considering the 50-fold dilution introduced during sample preparation, the calculated sample-based LODs remained well below the concentrations typically encountered in human whole blood, indicating that the proposed method provides sufficient sensitivity for routine biomonitoring applications. This analytical performance reflects the combined benefits of the microwave-assisted alkaline extraction procedure and the ICP-MS/MS operating conditions. Suitable calibration performance was obtained for all analytes over the selected concentration ranges, with regression coefficients (R) ≥ 0.999. Detailed calibration parameters, including the calibration levels used, regression equations, and regression coefficients, are provided in the Supplementary Materials (Table S1).

Moreover, the selected calibration ranges were designed to encompass the wide concentration differences among the investigated analytes in whole blood. S and Cl are major elements constituents present at g L^−1^ concentrations, Br occurs at intermediate mg L^−1^ levels, whereas B and I are typically found at considerably lower µg L^−1^ concentrations [44,45]. Despite these differences spanning approximately six orders of magnitude, the proposed ICP-MS/MS method enabled the simultaneous determination of all analytes using a single instrumental acquisition method, demonstrating the versatility of the proposed analytical approach.

To investigate the precision and accuracy of the proposed method, whole-blood proficiency testing materials, reference materials, and spiked whole-blood samples were analyzed in triplicate. The results are summarized in Table 3, Table 4, Table 5, Table 6 and Table 7. The precision obtained for B, Br, and I complied with the concentration-dependent acceptance criteria estimated according to the Horwitz equation (AOAC Appendix F) [46,47]. For S and Cl, however, some samples presented RSD values slightly above the Horwitz-predicted limits. This behavior is not unexpected, as the Horwitz equation was empirically derived from interlaboratory studies over a broad concentration range and predicts increasingly stringent precision requirements as analyte concentration increases. Consequently, for major elements such as S and Cl, which are present at g L^−1^ concentrations in whole blood, the predicted RSD values (approximately 3.5%) may underestimate the overall method uncertainty, as the Horwitz model does not account for the cumulative variability introduced by individual sample preparation, microwave-assisted alkaline extraction, matrix-related effects, and instrumental determination by ICP-MS/MS. In this context, the observed repeatability, with RSD values up to 15%, was considered acceptable for the intended application, particularly in view of the satisfactory agreement with available reference values and the spikes recoveries obtained. Furthermore, ICP-MS methods for elemental analysis in whole blood have reported repeatability values below 15% as suitable for biomonitoring applications, and clinical validation studies have established acceptance criteria of ≤20% CV for method precision [32,48].

Also, the results obtained for quality control and reference materials showed satisfactory agreement with the available reference values, while the spike-recovery experiments provided satisfactory recoveries, collectively supporting the trueness of the proposed procedure. The good agreement observed for both low- and high-concentration quality-control samples demonstrate that the method is suitable over the entire working concentration range. Furthermore, to the best of our knowledge, this is the first study to report B, Br, and I concentrations in NIST SRM 1577c determined by ICP-MS/MS. Although these values are not certified by NIST, they were obtained using a rigorously evaluated analytical method and may serve as useful comparative data for future analytical studies involving this reference material.

Another potential concern in whole-blood analysis is the relatively high matrix load introduced into the analytical system, which may influence nebulization efficiency and plasma ionization. To evaluate the effect of matrix load on the proposed method, samples containing reduced (LM = Less Matrix, 50 µL of whole blood) and increased (MM = More Matrix, 150 µL of whole blood) matrix amounts were compared with the routine preparation using 100 µL of whole blood. Relative biases were calculated using the routine preparation as the reference. For LM, relative biases were −12.0%, 6.3%, 7.3%, 5.6%, and 0.3% for B, S, Cl, Br, and I, respectively, whereas for MM, the corresponding values were −1.8%, −4.7%, −5.3%, −2.7%, and 3.3%. It should be noted that the B concentration obtained under the LM condition was below the applicable LOQ. Because only half of the routine sample volume was used for the LM preparation, the LOD and LOQ expressed in the original sample are approximately twofold higher than those established for the routine 100 µL preparation. Therefore, the −12.0% bias observed for B under the LM condition should be interpreted cautiously. Nevertheless, all relative biases observed under the evaluated matrix-load conditions were within ±15%, consistent with commonly applied acceptance criteria for bioanalytical methods [32,48,49]. No statistically significant differences were observed between the evaluated preparations using a two-tailed *t*-test (*p* > 0.05). Overall, these results indicate that the variation in matrix load evaluated in this study had a limited influence on the analytical results.

The short-term stability of the prepared analytical solutions was also evaluated. No statistically significant differences (*p* > 0.05) were observed between the initial (<8 h) and 24–36 h measurements. The results are summarized in Figure 1. Relative biases between the two measurement periods ranged from −9.9% to 14.8% across all analytes and samples, remaining within ±15%, consistent with commonly applied acceptance criteria for bioanalytical methods [32,48,49]. These results support the short-term stability of the prepared analytical solutions at room temperature for at least 24 h under the evaluated conditions. Detailed results are provided in the Supplementary Materials (Table S6).

Although several analytical techniques have been reported for the determination of non-metal elements in biological samples, most are dedicated to a single analyte or a limited group of analytes [2]. Ion-selective electrodes (ISEs) and ion chromatography (IC) are well-established techniques for the determination of halogens in clinical and environmental samples [2,27,50]. However, ISEs are dedicated to a single analyte, whereas IC is generally limited to inorganic ionic species unless complete conversion of organohalogen and organosulfur compounds into their corresponding inorganic ions is achieved during sample preparation. Consequently, these techniques do not directly provide the total elemental concentration if the sample preparation is not designed for that.

In contrast, atomic spectrometry techniques, including ICP-MS/MS and ICP-OES, overcome these limitations by providing total elemental determination regardless of the original chemical species, provided that an appropriate sample preparation procedure is employed [2]. For this reason, alkaline extraction has become one of the preferred approaches for the determination of halogens in biological samples because it preserves analyte stability, minimizes volatilization losses, and produces analytical solutions fully compatible with plasma-based techniques [2]. Combustion-based sample preparation methods, including microwave-induced combustion (MIC) [51,52,53] and the more recent microwave-induced combustion using disposable vials (MIC-DV) [45], have also demonstrated excellent performance for the determination of total halogens and sulfur in biological samples. Nevertheless, these methods still require specialized accessories, and additional operational steps before instrumental analysis. In contrast, the proposed method is based solely on microwave-assisted alkaline extraction performed directly in the same disposable polypropylene vial used for ICP-MS analysis, eliminating sample transfer steps and simplifying the analytical workflow.

Furthermore, Table 8 provides a comparison with representative ICP-MS-based methods previously reported for the determination of B, S, Cl, Br, and I in biological matrices. Although these elements have been determined individually or in different combinations, to the best of our knowledge, no previous study has reported the determination of all five elements in whole blood within a single ICP-MS method. As shown in Table 8, the reported approaches differ considerably in sample preparation, analyte coverage, and instrumental strategy. Several methods employ no-gas conditions, under which some of these elements, particularly S and Cl, may be susceptible to spectral interferences depending on the matrix and dilution applied, whereas other approaches rely on more extensive sample preparation procedures, including MIC or MAD-UV digestion. In comparison, the present method combines a small sample volume (100 µL) with a simple MAE method. The use of O_2_ enables mass-shift determination of S and Cl and contributes to spectral interference control, while monitoring Cl as ^35^Cl^18^O^+^ provides controlled signal attenuation. Together, these characteristics provide a streamlined approach for the determination of B, S, Cl, Br, and I from a single whole-blood aliquot, supporting its potential applicability to biomonitoring and large-scale studies.

## 4. Conclusions

A microwave-assisted alkaline extraction method was developed for the determination of B, S, Cl, Br, and I in whole blood by ICP-MS/MS. The proposed workflow requires only 100 µL of sample and performs sample preparation and instrumental analysis in the same disposable polypropylene vial, simplifying the analytical procedure while providing satisfactory analytical performance.

A key feature of the method is the use of a single tune for all analytes determination. The deliberate formation of ^35^Cl^18^O^+^ using the ^18^O isotope provided a practical strategy to attenuate the Cl signal, enabling the simultaneous determination of major and trace-level non-metal elements without detector saturation. To the best of our knowledge, this is the first report exploiting the formation of ^35^Cl^18^O^+^ to intentionally attenuate the Cl signal for ICP-MS/MS determination in biological samples.

The simplified sample preparation and relatively short instrumental analysis time facilitate the analysis of large sample batches, supporting the potential applicability of the method to biomonitoring and large-scale epidemiological studies. Furthermore, the analytical results obtained for NIST SRM 1577c provide additional reference data for B, Br, and I in this widely used biological reference material, helping to address the limited literature available for these analytes and supporting future analytical studies.

Overall, the proposed method provides a practical and reliable analytical workflow for the routine determination of non-metal elements in whole blood samples.

## Figures and Tables

**Figure 1:**
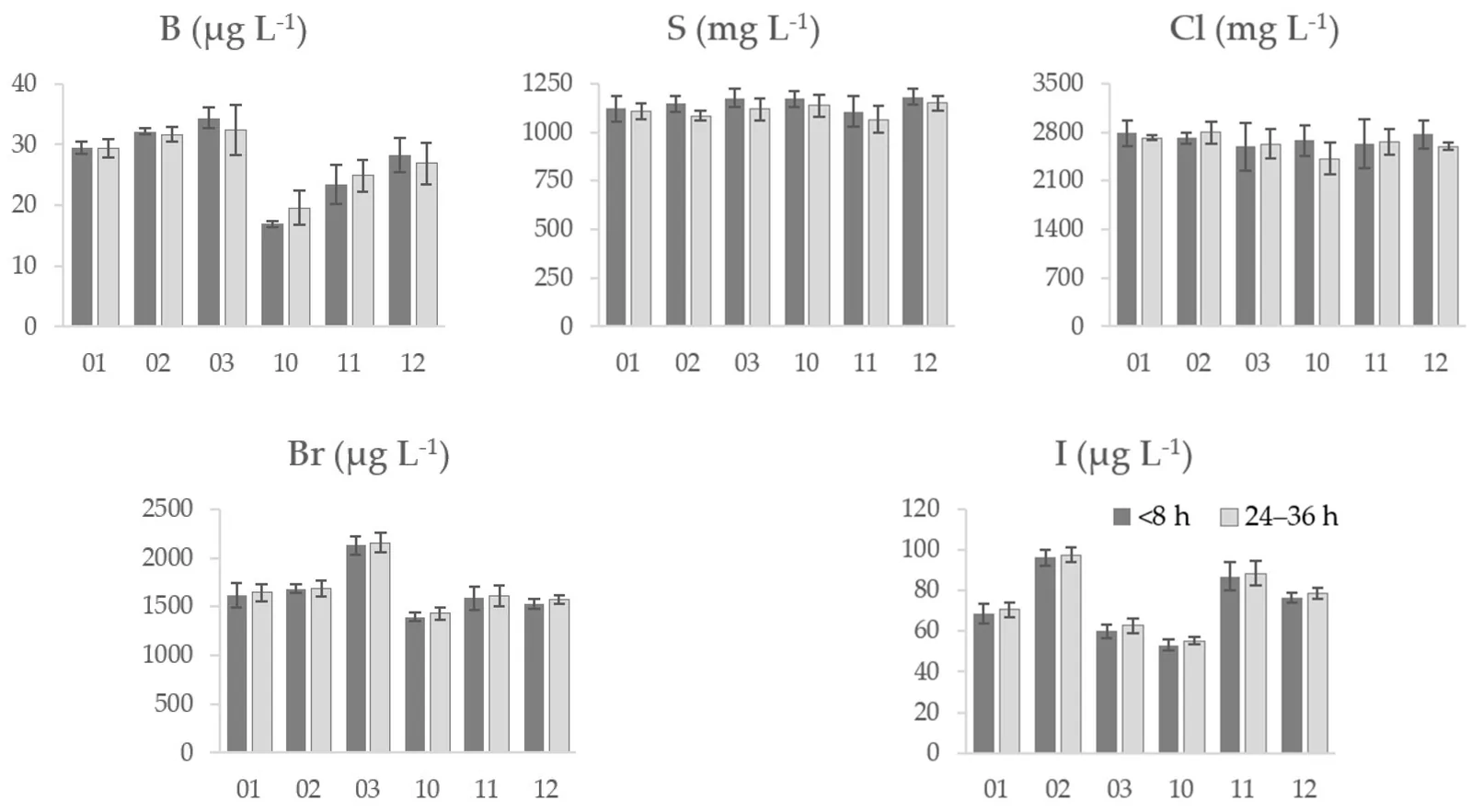
Short-term stability of analytical solutions at room temperature. Bars represent the mean ± SD (*n* = 3) for freshly prepared (<8 h) and stored (24–36 h) solutions of the QM-B-Q26xx proficiency testing materials (xx = 01, 02, 03, 10, 11, and 12). Each panel represents one analyte (B, S, Cl, Br, and I). Detailed results are provided in the Supplementary Materials.

**Table 1:** ICP-MS operating conditions.

Parameters	Value
RF Power	1550 W
Nebulizer gas	0.90 L min^−1^
Nebulizer	MicroMist^®^ 0.4 mL/min
Makeup gas	0.30 L min^−1^
Plasma option gas	5% (20% O_2_ in Ar)
Spray chamber	2 °C
Collision/reaction cell	0.25 mL min^−1^ O_2_
Acquisition mode	3 replicates, 30 sweeps/replicate
Q1 → Q2, dwell time	^10^B → ^10^B, m/z 10 (Monitoring), 0.10 s
	^11^B → ^11^B, m/z 11 (Target), 0.10 s
	^33^S → ^33^S^16^O, m/z 49 (Target), 0.10 s
	^35^Cl → ^35^Cl^18^O, m/z 53 (Target), 0.10 s
	^79^Br → ^79^Br, m/z 79 (Target), 0.15 s
	^127^I → ^127^I, m/z 127 (Target), 0.70 s
	^121^Sb → ^121^Sb, m/z 121 (ISTD), 0.10 s
	^193^Ir → ^193^Ir, m/z 193 (ISTD), 0.05 s

**Table 2:** LOD and LOQ expressed in the analytical solution and in whole blood sample.

Analyte	In Analytical Solution ^a^		In Sample ^a,b^	
	A-LOD	A-LOQ	S-LOD	S-LOQ
B (µg L^−1^)	0.121	0.398	6.03	19.9
S (mg L^−1^)	0.003	0.009	0.134	0.443
Cl (mg L^−1^)	0.488	1.61	24.4	80.5
Br (µg L^−1^)	0.125	0.413	6.26	20.7
I (µg L^−1^)	0.019	0.062	0.944	3.12

ᵃ Calculated from method blanks (*n* = 10); ᵇ S-LOD and S-LOQ values were converted to whole-blood concentrations using the 50-fold sample dilution factor.

**Table 3:** Results of boron (B) in evaluated samples (*n* = 3).

Sample	Result	Rec. (%)	Reference Values
	Mean ± SD (RSD, %)		
QC-1	39.3 ± 1.4 µg L^−1^ (3.5)	98.2	40
QC-2	400 ± 23 µg L^−1^ (5.8)	100.1	400
CRM-MP	<LOD	-	-
NIST SRM 1577c	169 ± 22 µg kg^−1^ (13)	99.1	170 (140–200) ^c^
QM-B-Q2601	29.4 ± 1 µg L^−1^ (3.3)	-	-
QM-B-Q2602	32.2 ± 0.5 µg L^−1^ (1.5)	-	-
QM-B-Q2603	34.4 ± 1.7 µg L^−1^ (4.8)	-	-
QM-B-Q2610	17 ± 0.5 µg L^−1^ (3.1) ^d^	-	-
QM-B-Q2611	23.4 ± 3.2 µg L^−1^ (13.5)	-	-
QM-B-Q2612	28.3 ± 2.8 µg L^−1^ (10)	-	-
QM-B-Q2603 (50% LM) ^a^	30.2 ± 3.4 µg L^−1^ (11.1) ^e^	-	-
QM-B-Q2603 (50% MM) ^a^	33.8 ± 2.1 µg L^−1^ (6.2)	-	-
Spike Test (in QM-B-Q2603) ^b^	1811 µg L^−1^	103.5	1750

^a^ LM = Less Matrix and MM = More Matrix; ^b^ See Supplementary Materials (Table S5) for more details; ^c^ Assigned values obtained from the Wadsworth Center interlaboratory study using NIST SRM 1577c; these values are not NIST-certified; ^d^ Value between the LOD and LOQ; ^e^ Value between the LOD and LOQ for the LM condition (considering the 100-fold sample dilution factor).

**Table 4:** Results of sulfur (S) in evaluated samples (*n* = 3).

Sample	Result	Rec. (%)	Reference Values
	Mean ± SD (RSD, %)		
QC-1	2.87 ± 0.04 mg L^−1^ (1.3)	95.7	3
QC-2	29.5 ± 0.4 mg L^−1^ (1.4)	98.3	30
CRM-MP	33.2 ± 0.3 mg L^−1^ (0.9)	94.7	35
NIST SRM 1577c	7516 ± 198 mg kg^−1^ (2.6)	100.3	7490 (7150–7830)
QM-B-Q2601	1120 ± 65 mg L^−1^ (5.8)	-	-
QM-B-Q2602	1145 ± 40 mg L^−1^ (3.5)	-	-
QM-B-Q2603	1174 ± 47 mg L^−1^ (4)	-	-
QM-B-Q2610	1172 ± 41 mg L^−1^ (3.5)	-	-
QM-B-Q2611	1106 ± 77 mg L^−1^ (7)	-	-
QM-B-Q2612	1181 ± 40 mg L^−1^ (3.4)	-	-
QM-B-Q2603 (50% LM) ^a^	1249 ± 60 mg L^−1^ (4.8)	-	-
QM-B-Q2603 (50% MM) ^a^	1119 ± 37 mg L^−1^ (3.3)	-	-
Spike Test (in QM-B-Q2603) ^b^	699 mg L^−1^	93.2	750

^a^ LM = Less Matrix and MM = More Matrix; ^b^ See Supplementary Materials (Table S5) for more details.

**Table 5:** Results of chlorine (Cl) in evaluated samples (*n* = 3).

Sample	Result	Rec. (%)	Reference Values
	Mean ± SD (RSD, %)		
QC-1	29 ± 1.4 mg L^−1^ (4.8)	96.8	30
QC-2	313 ± 5 mg L^−1^ (1.7)	104.3	300
CRM-MP	94.9 ± 2.6 mg L^−1^ (2.7)	94.9	100
NIST SRM 1577c	2949 ± 56 mg kg^−1^ (1.9)	102.8	2870 (2740–3000)
QM-B-Q2601	2785 ± 182 mg L^−1^ (6.5)	-	-
QM-B-Q2602	2716 ± 83 mg L^−1^ (3)	-	-
QM-B-Q2603	2591 ± 340 mg L^−1^ (13.1)	-	-
QM-B-Q2610	2681 ± 224 mg L^−1^ (8.3)	-	-
QM-B-Q2611	2637 ± 355 mg L^−1^ (13.5)	-	-
QM-B-Q2612	2769 ± 201 mg L^−1^ (7.3)	-	-
QM-B-Q2603 (50% LM) ^a^	2780 ± 305 mg L^−1^ (11)	-	-
QM-B-Q2603 (50% MM) ^a^	2453 ± 72 mg L^−1^ (2.9)	-	-
Spike Test (in QM-B-Q2603) ^b^	1119 mg L^−1^	111.9	1000

^a^ LM = Less Matrix and MM = More Matrix; ^b^ See Supplementary Materials (Table S5) for more details.

**Table 6:** Results of bromine (Br) in evaluated samples (*n* = 3).

Sample	Result	Rec. (%)	Reference Values
	Mean ± SD (RSD, %)		
QC-1	30.4 ± 0.3 µg L^−1^ (1)	101.2	30
QC-2	306 ± 2 µg L^−1^ (0.7)	102.1	300
CRM-MP	99.3 ± 0.8 µg L^−1^ (0.9)	99.3	100
NIST SRM 1577c	11,485 ± 558 µg kg^−1^ (4.9)	114.8	10,000 (5600–14,400) ^c^
QM-B-Q2601	1612 ± 127 µg L^−1^ (7.9)	-	-
QM-B-Q2602	1685 ± 49 µg L^−1^ (2.9)	-	-
QM-B-Q2603	2128 ± 95 µg L^−1^ (4.4)	-	-
QM-B-Q2610	1395 ± 45 µg L^−1^ (3.2)	-	-
QM-B-Q2611	1585 ± 122 µg L^−1^ (7.7)	-	-
QM-B-Q2612	1528 ± 52 µg L^−1^ (3.4)	-	-
QM-B-Q2603 (50% LM) ^a^	2248 ± 113 µg L^−1^ (5)	-	-
QM-B-Q2603 (50% MM) ^a^	2071 ± 78 µg L^−1^ (3.8)	-	-
Spike Test (in QM-B-Q2603) ^b^	968 µg L^−1^	96.8	1000

^a^ LM = Less Matrix and MM = More Matrix; ^b^ See Supplementary Materials (Table S5) for more details; ^c^ Assigned values obtained from the Wadsworth Center interlaboratory study using NIST SRM 1577c; these values are not NIST-certified.

**Table 7:** Results of iodine (I) in evaluated samples (*n* = 3).

Sample	Result	Rec. (%)	Reference Values
	Mean ± SD (RSD, %)		
QC-1	2.94 ± 0.02 µg L^−1^ (0.6)	97.9	3
QC-2	29.9 ± 0.3 µg L^−1^ (1.2)	99.8	30
CRM-MP	<LOD	-	-
NIST SRM 1577c	191 ± 8 µg kg^−1^ (4.1)	98.0	195 ^c^
QM-B-Q2601	68.7 ± 4.8 µg L^−1^ (7)	93.8	73.3 (57.6–89)
QM-B-Q2602	96.2 ± 3.7 µg L^−1^ (3.9)	94.3	102 (80.6–123)
QM-B-Q2603	60.1 ± 3.2 µg L^−1^ (5.4)	94.1	63.9 (50–77.8)
QM-B-Q2610	53.2 ± 2.6 µg L^−1^ (4.9)	97.7	54.4 (43.1–65.7)
QM-B-Q2611	86.9 ± 6.9 µg L^−1^ (7.9)	96.0	90.6 (71.8–109)
QM-B-Q2612	76.5 ± 2.5 µg L^−1^ (3.3)	97.0	78.8 (62.4–95.2)
QM-B-Q2603 (50% LM) ^a^	60.3 ± 3.4 µg L^−1^ (5.6)	94.4	63.9 (50–77.8)
QM-B-Q2603 (50% MM) ^a^	62.1 ± 1.9 µg L^−1^ (3)	97.2	63.9 (50–77.8)
Spike Test (in QM-B-Q2603) ^b^	103 µg L^−1^	103.0	100

^a^ LM = Less Matrix and MM = More Matrix; ^b^ See Supplementary Materials (Table S5) for more details; ^c^ Value obtained during the Wadsworth Center interlaboratory study using NIST SRM 1577c; this value is not NIST-certified.

**Table 8:** Comparison of the proposed method with representative ICP-MS-based methods for the determination of B, S, Cl, Br, and I in biological matrices.

Matrix	Element(s)	Sample Preparation ^a^	Instrument	Ref.
Whole Blood	B, S, Cl, Br, I ISTD: Sb	Prep.: 0.1 mL whole blood by MAE at 90 °C using TMAH and 0.1% EDTA; DF: 50×.	ICP-QQQ; O_2_ mode	This work
Whole blood	Br, Cl, I ISTD: None	Prep.: 100–500 mg blood by MIC using water or NH_4_OH; alternatively, 150 mg by MAD-UV using HNO_3_/H_2_O_2_.	ICP-MS No-gas mode	[51]
Whole blood and hair	B; ISTD: Y, Rh	Prep.: 0.5 mL whole blood and NR mass hair by MAD using HNO_3_/H_2_O_2_.	ICP-MS DRC (NH_3_)	[54]
Serum, plasma and whole blood	P, S ISTD: Rh	Prep.: Simple dilution using 1% HNO_3_ and 2% ethanol, DF: NR.	ICP-QQQ O_2_ mode	[55]
Saliva	Br, I; ISTD: None	Prep.: 0.5–2 mL saliva by MAE at 90 °C using TMAH/NH_4_OH; DF: 10–40×; alternatively, 0.05–0.3 mL by MIC using NH_4_OH or water; DF: 67–400×.	ICP-MS No-gas mode	[42]
Serum	I, Br; ISTD: Te	Prep.: Simple dilution 1:10 and vortex homogenization using 1% TMAH and 0.01% Triton X-100; DF: 10×.	ICP-MS No-gas mode	[56]
Nails	I, Br; ISTD: None	Prep.: 100 mg nails by MAE at 90 °C using TMAH/NH_4_OH, DF: 200×; alternatively, 30–100 mg by MIC using NH_4_OH, DF: 200–666×.	ICP-MS No-gas mode	[43]
Hair	I, Br; ISTD: None	Prep.: 300 mg hair by MAE at 90 °C using TMAH/NH_4_OH, DF: 66×; alternatively, 50–400 mg by MIC using NH_4_OH/water, DF: 50–400×.	ICP-MS No-gas mode	[41]

ᵃ Prep., sample preparation; DF, dilution factor; MAE, microwave-assisted extraction; MIC, microwave-induced combustion; MAD, microwave-assisted digestion; MAD-UV, microwave-assisted ultraviolet digestion; NR, not reported.

## Data Availability

The original contributions presented in this study are included in the article/Supplementary Materials. Further inquiries can be directed to the corresponding author.

## Supplementary Materials

The following supporting information can be downloaded at https://www.mdpi.com/article/10.3390/toxics14100859/s1, Table S1: Calibration parameters for the determination of B, S, Cl, Br, and I by ICP-MS/MS; Table S2: Calibration parameters for non-metal determination using Sb and Ir as internal standards; Table S3: Limits of detection and quantification in whole blood using Sb and Ir as internal standards; Table S4: Comparison of results obtained using Sb and Ir as internal standards (ISTDs); Table S5: Precision and recovery results for spiked whole blood proficiency testing material (QM-B-Q2603); Table S6: Evaluation of the short-term stability of analytical solutions for B, S, Cl, Br, and I; Table S7: Comparison of ^35^Cl^16^O^+^ and ^35^Cl^18^O^+^ sensitivity; Table S8: Evaluation of potential ^40^Ar^39^K^+^ interference on ^79^Br^+^ determination in whole-blood sample under O_2_ cell conditions [57,58].

## Abbreviations

The following abbreviations are used in this manuscript:

B	Boron
Br	Bromine
Cl	Chlorine
Dil.	Diluent
DF	Dilution Factor
I	Iodine
Ir	Iridium
ICP-MS	Inductively Coupled Plasma Mass Spectrometry
ICP-QQQ	Triple Quadrupole ICP-MS
ISTD	Internal Standard
LDL	Low Density Lipoprotein-cholesterol
LOD	Limit of detection
LOQ	Limit of quantification
MM	More Matrix
LM	Less Matrix
MAD	Microwave-Assisted Digestion
MAD-UV	Microwave-Assisted Ultraviolet Digestion
MAE	Microwave-Assisted Extraction
MIC	Microwave-Induced Combustion
NR	Not Reported
Prep.	Sample Preparation
Rec.	Recovery
RSD	Relative Standard Deviation
S	Sulfur
Sb	Antimony
SD	Standard Deviation
TMAH	Tetramethylammonium Hydroxide
VLDL	Very-Low-Density Lipoprotein-cholesterol
