# Supplementary material for: A Simple Microwave-Assisted Method for the Determination of Boron, Sulfur, Bromine, Chlorine, and Iodine in Whole Blood by ICP-QQQ for Biomonitoring Applications
Source: Toxics. 2026 Sep 28;14(10):859. doi: 10.3390/toxics14100859 (PMC13709887; doi:10.3390/toxics14100859)
Supplement: Supplementary file 1 [file toxics-14-00859-s001.zip › toxics-4538472-supplementary.pdf]

Supplementary Materials

A Simple Microwave-Assisted Method for the Determination of Boron, Sulfur, Bromine, Chlorine, and Iodine in Whole Blood by ICP-QQQ for Biomonitoring Applications

Lucas Schmidt [1], Alma Malibekova [2], Thieli Schaefer Nunes [1], Kayla Peterson [1], Rosalind Wright [1] and Julio Alberto Landero-Figueroa [1,*]

Table of contents

No.	Legend
Table S1	Calibration parameters for the determination of B, S, Cl, Br, and I by ICP-MS/MS.
Table S2	Calibration parameters for non-metal determination using Sb and Ir as internal standards.
Table S3	Limits of detection and quantification in whole blood using Sb and Ir as internal standards.
Table S4	Comparison of results obtained using Sb and Ir as internal standards (ISTDs).
Table S5	Precision and recovery results for spiked whole blood proficiency testing material (QM-B-Q2603).
Table S6	Evaluation of the short-term stability of analytical solutions for B, S, Cl, Br, and I.
Table S7	Comparison of $^{35}Cl^{16}O^+$ and $^{35}Cl^{18}O^+$ sensitivity.
Table S8	Evaluation of potential $^{40}Ar^{39}K^+$ interference on $^{79}Br^+$ determination in whole-blood sample under O_2 cell conditions.

Note 1: Unless otherwise specified, results reported as n = 3 throughout the manuscript and Supplementary Materials represent independently prepared replicates analyzed within the same analytical batch/day.

Note 2: B is a metalloid; for simplicity, it is included within the term "non-metals" throughout this study.

S1. Calibration parameters for the determination of B, S, Cl, Br, and I by ICP-MS/MS.

Calibration curves were constructed using at least five calibration levels selected from the seven standards prepared for each analyte. The levels included in each regression were selected based on the concentration range observed in the analyzed samples to provide an appropriate working range for each analyte. Table S1 summarizes the calibration ranges used for regression, analytical transitions, regression equations, correlation coefficients (R), and relative standard errors (%RSE). All calibration curves were fitted using weighted (1/y) linear regression without forcing the regression through the origin, with ^{121}Sb used as the internal standard.

Table S1. Calibration parameters for the determination of B, S, Cl, Br, and I by ICP-MS/MS.

Element	Transition	Calibration Range	Calibration levels used (μg L^{-1})	Equation*	R	%RSE
10B (monitoring)	10 → 10	0.5–100 μg L^{-1}	0.5, 5, 10, 50, 100	Y = 4.497922E-04 X + 0.000105	0.99997	3.30
B (analyte)	11 → 11	0.5–100 μg L^{-1}	0.5, 5, 10, 50, 100	Y = 2.398884E-03 X + 0.000450	0.99991	1.30
S (analyte)	33 → 49	500–50,000 μg L^{-1}	500, 1000, 5000, 10,000, 20,000, 50,000	Y = 1.349821E-05 X + 0.001608	0.99998	2.96
Cl (analyte)	35 → 53	5000–200,000 μg L^{-1}	5000, 10,000, 50,000, 100,000, 200,000	Y = 5.010135E-09 X + 0.000018	0.99983	3.81
Br (analyte)	79 → 79	0.5–200 μg L^{-1}	0.5, 5, 10, 50, 100, 200	Y = 1.039613E-04 X + 0.000020	0.99986	2.38
I (analyte)	127 → 127	0.05–20 μg L^{-1}	0.05, 0.5, 1, 5, 10, 20	Y = 3.965533E-03 X + 0.000650	0.99995	3.38

* Calibration curves were fitted using weighted (1/y) linear regression without forcing the regression through the origin; ^{121}Sb was used as the internal standard (ISTD).

S2. Comparison of Sb and Ir as Internal Standards (ISTD)

To further evaluate the suitability of the internal standard used in the proposed method, the analytical data were processed using either Sb or Ir as the internal standard for B, S, Cl, Br, and I. As shown in Tables S2 to S4, both internal standards provided suitable correction across all analytes. The concentrations obtained using Ir showed relative biases of 4.2–15.3% compared with those obtained using Sb, while comparable S-LOD and S-LOQ values were obtained with both internal standards. Moreover, the highest relative biases were observed for samples with analyte concentrations below or near the LOQ, where greater variability is expected. Therefore, based on these results, Sb was maintained as the primary internal standard for the proposed method, while Ir was considered a suitable alternative.

Table S2. Calibration parameters for non-metal determination using Sb and Ir as internal standards.

Element	Transition	Equation*	R	%RSE
**Using 121Sb as ISTD**				
10B (monitoring)	10 → 10	Y = 4.497922E-04 X + 0.000105	0.99997	3.30
B (analyte)	11 → 11	Y = 2.398884E-03 X + 0.000450	0.99991	1.30
S (analyte)	33 → 49	Y = 1.349821E-05 X + 0.001608	0.99998	2.96
Cl (analyte)	35 → 53	Y = 5.010135E-09 X + 0.000018	0.99983	3.81
Br (analyte)	79 → 79	Y = 1.039613E-04 X + 0.000020	0.99986	2.38
I (analyte)	127 → 127	Y = 3.965533E-03 X + 0.000650	0.99995	3.38
**Using 193Ir as ISTD**				
10B (monitoring)	10 → 10	Y = 8.955802E-04 X + 0.000210	0.99996	3.47
B (analyte)	11 → 11	Y = 4.776409E-03 X + 0.000901	0.99990	1.28
S (analyte)	33 → 49	Y = 2.639351E-05 X + 0.003818	0.99998	1.41
Cl (analyte)	35 → 53	Y = 9.849685E-09 X + 0.000038	0.99996	2.39
Br (analyte)	79 → 79	Y = 2.048072E-04 X + 0.000046	0.99997	2.14
I (analyte)	127 → 127	Y = 7.807955E-03 X + 0.001333	0.99998	1.53

* Calibration curves were fitted using weighted (1/y) linear regression without forcing the regression through the origin.

Table S3. Limits of detection and quantification in whole blood using Sb and Ir as internal standards.

Analyte	Using Sb as ISTD [a,b]		Using Ir as ISTD [a,b]	
	S-LOD	S-LOQ	S-LOD	S-LOQ
10B (monitoring) (μg L^{-1})	8.23	27.2	8.59	28.3
B (μg L^{-1})	6.03	19.9	6.30	20.8
S (mg L^{-1})	0.134	0.443	0.199	0.656
Cl (mg L^{-1})	24.4	80.5	24.0	79.2
Br (μg L^{-1})	6.26	20.7	6.51	21.5
I (μg L^{-1})	0.944	3.12	1.02	3.37

[a] Calculated from method blanks (n = 10); [b] S-LOD and S-LOQ values were converted to whole-blood concentrations using the 50-fold sample dilution factor.

Table S4. Comparison of results obtained using Sb and Ir as internal standards (ISTDs).

Sample	Mean ± SD (RSD, %), *n* = 3		Relative Bias (%)[a]
	using Sb as ISTD	using Ir as ISTD	
**10B - Monitoring (µg L^{-1})**			
QM-B-Q2601	30.1 ± 3 (10)	32.3 ± 3.2 (10)	7.3
QM-B-Q2602	34.2 ± 4.4 (12.8)	36.4 ± 4.5 (12.5)	6.4
QM-B-Q2603	31.9 ± 0.4 (1.2)	34.6 ± 0.3 (0.8)	8.5
QM-B-Q2610	17.3 ± 4.8 (27.9) [b]	19.9 ± 5.5 (27.6) [b]	15.0
QM-B-Q2611	26.9 ± 3.6 (13.3)	30.1 ± 3.9 (12.8)	11.9
QM-B-Q2612	31.7 ± 2.6 (8.3)	35.5 ± 2.8 (7.9)	12.0
**B (µg L^{-1})**			
QM-B-Q2601	29.4 ± 1 (3.3)	31.6 ± 1.1 (3.5)	7.5
QM-B-Q2602	32.2 ± 0.5 (1.5)	34.3 ± 0.5 (1.5)	6.5
QM-B-Q2603	34.4 ± 1.7 (4.8)	37.3 ± 2.2 (5.9)	8.4
QM-B-Q2610	17.0 ± 0.5 (3.1) [b]	19.6 ± 1 (4.9) [b]	15.3
QM-B-Q2611	23.4 ± 3.2 (13.5)	26.7 ± 3.8 (14.2)	14.1
QM-B-Q2612	28.3 ± 2.8 (10)	31.9 ± 2.9 (9)	12.7
**S (mg L^{-1})**			
QM-B-Q2601	1120 ± 65 (5.8)	1172 ± 71 (6)	4.6
QM-B-Q2602	1145 ± 40 (3.5)	1193 ± 35 (2.9)	4.2
QM-B-Q2603	1174 ± 47 (4)	1237 ± 55 (4.5)	5.4
QM-B-Q2610	1172 ± 41 (3.5)	1250 ± 55 (4.4)	6.7
QM-B-Q2611	1106 ± 77 (7)	1180 ± 84 (7.1)	6.7
QM-B-Q2612	1181 ± 40 (3.4)	1265 ± 43 (3.4)	7.1
**Cl (mg L^{-1})**			
QM-B-Q2601	2785 ± 182 (6.5)	2944 ± 196 (6.7)	5.7
QM-B-Q2602	2716 ± 83 (3)	2862 ± 89 (3.1)	5.4
QM-B-Q2603	2591 ± 340 (13.1)	2761 ± 358 (13)	6.6
QM-B-Q2610	2681 ± 224 (8.3)	2895 ± 256 (8.8)	8.0
QM-B-Q2611	2637 ± 355 (13.5)	2848 ± 371 (13)	8.0
QM-B-Q2612	2769 ± 201 (7.3)	3002 ± 218 (7.3)	8.4
**Br (µg L^{-1})**			
QM-B-Q2601	1612 ± 127 (7.9)	1698 ± 138 (8.1)	5.3
QM-B-Q2602	1685 ± 49 (2.9)	1769 ± 42 (2.4)	5.0
QM-B-Q2603	2128 ± 95 (4.4)	2258 ± 114 (5)	6.1
QM-B-Q2610	1395 ± 45 (3.2)	1499 ± 61 (4.1)	7.5
QM-B-Q2611	1585 ± 122 (7.7)	1704 ± 134 (7.9)	7.5
QM-B-Q2612	1528 ± 52 (3.4)	1649 ± 56 (3.4)	7.9
**I (µg L^{-1})**			
QM-B-Q2601	68.7 ± 4.8 (7)	72.7 ± 5.3 (7.2)	5.8
QM-B-Q2602	96.2 ± 3.7 (3.9)	101.2 ± 3.3 (3.3)	5.2
QM-B-Q2603	60.1 ± 3.2 (5.4)	64.3 ± 3.7 (5.7)	7.0
QM-B-Q2610	53.2 ± 2.6 (4.9)	57.8 ± 3.4 (5.9)	8.6
QM-B-Q2611	86.9 ± 6.9 (7.9)	94.1 ± 7.5 (7.9)	8.3
QM-B-Q2612	76.5 ± 2.5 (3.3)	83.2 ± 2.6 (3.2)	8.8

[a] Relative bias was calculated using the Sb-corrected results as the reference; [b] Value between the LOD and LOQ.

S3. Precision and recovery results for spiked whole blood proficiency testing material (QM-B-2603).

Table S5. Precision and recovery results for spiked whole blood proficiency testing material (QM-B-Q2603).

Analyte	Spike value	Measured value[a]			Recovery (%)	Acceptance RSD (%)[b]
		Unspiked sample	Spiked sample	Spike value		
B (ug/L)	1750	34.4 ± 1.7 (4.8)	1845 ± 14 (0.8)	1811	103.5	9.7
S (mg/L)	750	1174 ± 47 (4)	1873 ± 49 (2.6)	699	93.2	3.4
Cl (mg/L)	1000	2591 ± 340 (13.1)	3710 ± 27 (0.7)	1119	111.9	3.1
Br (ug/L)	1000	2128 ± 95 (4.4)	3097 ± 155 (5)	968	96.8	9.0
I (ug/L)	100	60.1 ± 3.2 (5.4)	163 ± 4 (2.2)	103	103.0	14.0

[a]Results expressed as Mean ± SD (RSD, %) for $n = 3$; [b]Acceptable requirements were obtained according to the Horwitz equation.

S4. Evaluation of the short-term stability of analytical solutions for B, S, Cl, Br, and I.

As shown in Tables S6, no statistically significant differences were observed between the initial (<8 h) and 24–36 h measurements ($p > 0.05$). In addition, the relative biases between the two measurement periods ranged from −9.9% to 14.8% across all analytes and samples, remaining within ±15%. These results support the short-term stability of the analytical solutions for up to 36 h at room temperature.

Table S6. Evaluation of the short-term stability of analytical solutions for B, S, Cl, Br, and I.

Sample	Mean ± SD (RSD, %), *n* = 3		p-value	Relative Bias (%)
	<8 h	24–36 h		
**B (μg L⁻¹)**				
QM-B-Q2601	29.4 ± 1 (3.3)	29.3 ± 1.5 (5.3)	0.920	−0.4
QM-B-Q2602	32.2 ± 0.5 (1.5)	31.7 ± 1.3 (4.3)	0.618	−1.4
QM-B-Q2603	34.4 ± 1.7 (4.8)	32.4 ± 4.2 (13.1)	0.492	−5.7
QM-B-Q2610	17 ± 0.5 (3.1) [b]	19.6 ± 2.8 (14.4) [b]	0.199	14.8
QM-B-Q2611	23.4 ± 3.2 (13.5)	24.9 ± 2.6 (10.3)	0.561	6.4
QM-B-Q2612	28.3 ± 2.8 (10)	26.9 ± 3.4 (12.5)	0.627	−4.7
**S (mg L⁻¹)**				
QM-B-Q2601	1,120 ± 65 (5.8)	1,106 ± 43 (3.8)	0.772	−1.2
QM-B-Q2602	1,145 ± 40 (3.5)	1,085 ± 26 (2.4)	0.095	−5.2
QM-B-Q2603	1,174 ± 47 (4)	1,117 ± 58 (5.2)	0.257	−4.9
QM-B-Q2610	1,172 ± 41 (3.5)	1,136 ± 58 (5.1)	0.434	−3.0
QM-B-Q2611	1,106 ± 77 (7)	1,066 ± 70 (6.6)	0.542	−3.6
QM-B-Q2612	1,181 ± 40 (3.4)	1,150 ± 37 (3.2)	0.382	−2.6
**Cl (mg L⁻¹)**				
QM-B-Q2601	2,785 ± 182 (6.5)	2,720 ± 42 (1.5)	0.580	−2.3
QM-B-Q2602	2,716 ± 83 (3)	2,792 ± 157 (5.6)	0.500	2.8
QM-B-Q2603	2,591 ± 340 (13.1)	2,628 ± 211 (8)	0.879	1.4
QM-B-Q2610	2,681 ± 224 (8.3)	2,415 ± 227 (9.4)	0.222	−9.9
QM-B-Q2611	2,637 ± 355 (13.5)	2,664 ± 185 (6.9)	0.914	1.0
QM-B-Q2612	2,769 ± 201 (7.3)	2,590 ± 54 (2.1)	0.210	−6.5
**Br (μg L⁻¹)**				
QM-B-Q2601	1,612 ± 127 (7.9)	1,642 ± 87 (5.3)	0.753	1.9
QM-B-Q2602	1,685 ± 49 (2.9)	1,687 ± 78 (4.6)	0.975	0.1
QM-B-Q2603	2,128 ± 95 (4.4)	2,153 ± 100 (4.7)	0.773	1.2
QM-B-Q2610	1,395 ± 45 (3.2)	1,429 ± 63 (4.4)	0.485	2.5
QM-B-Q2611	1,585 ± 122 (7.7)	1,613 ± 104 (6.4)	0.779	1.8
QM-B-Q2612	1,528 ± 52 (3.4)	1,569 ± 42 (2.7)	0.338	2.7
**I (μg L⁻¹)**				
QM-B-Q2601	68.7 ± 4.8 (7)	70.6 ± 3.8 (5.3)	0.623	2.7
QM-B-Q2602	96.2 ± 3.7 (3.9)	97.5 ± 3.6 (3.7)	0.669	1.4
QM-B-Q2603	60.1 ± 3.2 (5.4)	62.7 ± 3.6 (5.7)	0.401	4.3
QM-B-Q2610	53.2 ± 2.6 (4.9)	55.2 ± 1.9 (3.4)	0.332	3.9

Sample	Mean ± SD (RSD, %), n = 3		p-value	Relative Bias (%)
	<8 h	24–36 h		
QM-B-Q2611	86.9 ± 6.9 (7.9)	88.5 ± 6.2 (7.1)	0.791	1.7
QM-B-Q2612	76.5 ± 2.5 (3.3)	78.7 ± 2.8 (3.5)	0.368	2.9

*Statistical significance was assessed between the <8 h and 24–36 h storage periods; $p > 0.05$ indicates no statistically significant difference; Relative bias was calculated using the initial measurement (<8 h after preparation) as the reference; [b] Value between the LOD and LOQ.

S5. Comparison of $^{35}Cl^{16}O^+$ and $^{35}Cl^{18}O^+$ sensitivity

To experimentally evaluate the difference in sensitivity between $^{35}Cl^{16}O^+$ and $^{35}Cl^{18}O^+$, both product ions were monitored using the same set of Cl calibration standards by including an additional acquisition channel for $^{35}Cl^{16}O^+$. The relative sensitivities were evaluated by comparing the slopes of the respective calibration curves.

As shown in Table S7, the calibration slopes were 1.409593E-06 for $^{35}Cl^{16}O^+$ and 4.723823E-09 for $^{35}Cl^{18}O^+$. The relative attenuation was calculated from the ratio of the calibration sensitivities:

$$Attenuation\,factor = \frac{slope_{35Cl16O+}}{slope_{35Cl18+}} = \frac{1.409593 \times 10^{-6}}{4.723823 \times 10^{-9}} = 298 \approx 300$$

Thus, monitoring $^{35}Cl^{18}O^+$ resulted in an approximately 300-fold reduction in sensitivity compared with $^{35}Cl^{16}O^+$. This experimentally observed attenuation is consistent with the substantially lower natural abundance of ^{18}O relative to ^{16}O, for which an approximately 500-fold difference would be expected based solely on their natural isotopic abundances. The experimental and theoretical attenuation factors are therefore of the same order of magnitude.

Table S7. Comparison of $^{35}Cl^{16}O^+$ and $^{35}Cl^{18}O^+$ sensitivity.

Element	Transition	Calibration range	Equation	R	%RSE
Cl	35 → 51 ($^{35}Cl^{16}O^+$)	5000 – 200,000 μg L^{-1}	Y = 1.409593E-06 X + 0.003446	0.99973	3.84
Cl	35 → 53 ($^{35}Cl^{18}O^+$)	5000 – 200,000 μg L^{-1}	Y = 4.723823E-09 X + 0.000019	0.99980	5.47

* Calibration curves were fitted using weighted (1/y) linear regression without forcing the regression through the origin; ^{121}Sb was used as the internal standard (ISTD); Calibration levels used = 5000, 10,000, 50,000, 100,000, 200,000 μg L^{-1}.

S6. Evaluation of potential K interference on $^{79}Br^+$ determination

The potential formation of $^{40}Ar^{39}K^+$ represents an important consideration for the on-mass determination of $^{79}Br^+$ in whole blood because of the relatively high concentration of potassium (K) in this matrix. In addition to endogenous K, further K may be introduced when blood is collected in K_2EDTA-containing tubes. The International Council for Standardization in Haematology recommends 1.5–2.2 mg $K_2EDTA{\cdot}2H_2O$ per mL of blood [57]. At the upper recommended concentration of 2.2 mg mL^{-1}, this corresponds to approximately 425 mg L^{-1} K contributed by the anticoagulant. Total elemental K concentrations of approximately 1,922–2,108 mg L^{-1} have been reported for human whole blood [58]. Thus, considering both endogenous K and the maximum contribution from K_2EDTA, a total K concentration of approximately 2,533 mg L^{-1} represents a reasonable high-end estimate for K_2EDTA-collected whole blood.

To experimentally evaluate the potential contribution of $^{40}Ar^{39}K^+$ to the $^{79}Br^+$ signal under the O_2 cell conditions employed in this method, aliquots of whole blood (QM-B-Q2610) were fortified with additional K at 1000, 1500, and 3000 mg L^{-1} using a commercial K standard (10,000 mg L^{-1}, CGK10, Inorganic Ventures, Christiansburg, VA, USA). These concentrations represent K added in addition to the endogenous K already present in the whole-blood sample, thereby producing deliberately elevated total K concentrations and providing a conservative challenge for potential $^{40}Ar^{39}K^+$ interference.

Table S8. Evaluation of potential $^{40}Ar^{39}K^+$ interference on $^{79}Br^+$ determination in whole-blood sample under O_2 cell conditions.

Sample	K added to whole blood (mg L^{-1})	Br found (μg L^{-1}), mean ± SD (n = 3)	Relative bias (%)*
Unspiked (QM-B-2610)	0	1437 ± 35	0.0
Spiked-level-1 (in QM-B-2610)	1,000	1431 ± 34	-0.4
Spiked-level-2 (in QM-B-2610)	1,500	1433 ± 27	-0.3
Spiked-level-3 (in QM-B-2610)	3,000	1426 ± 16	-0.7

* Relative bias was calculated using the unspiked sample as the reference within the same analytical sequence.

As shown in Table S8, the measured Br concentration remained essentially unchanged with increasing K addition. Relative biases compared with the unspiked sample were −0.4%, −0.3%, and −0.7% following the addition of 1000, 1500, and 3000 mg L^{-1} K, respectively. Even at the highest K addition, corresponding to an estimated total K concentration of approximately 5,000 mg L^{-1} in whole blood, the relative bias was only −0.7%. These results demonstrate that $^{40}Ar^{39}K^+$ did not produce a measurable interference on $^{79}Br^+$ determination under the O_2 cell conditions employed in this method, even at the deliberately elevated K concentrations evaluated.

References

57. U.S. Food and Drug Administration. BD Vacutainer® K2EDTA Blood Collection Tubes: 510(k) Summary; U.S. Food and Drug Administration: Silver Spring, MD, USA, 2024. Available online: FDA 510(k) Summary (accessed on 08 September 2026).
58. Harrington, J.M.; Young, D.J.; Essader, A.S.; Sumner, S.J.; Levine, K.E. Analysis of human serum and whole blood for mineral content by ICP-MS and ICP-OES: development of a mineralomics method. Biol Trace Elem Res 2014, 160, 132–142, doi:10.1007/s12011-014-0033-5.
